# Assisting Decision-Making on Age of Neutering for Mixed Breed Dogs of Five Weight Categories: Associated Joint Disorders and Cancers

**DOI:** 10.3389/fvets.2020.00472

**Published:** 2020-07-31

**Authors:** Benjamin L. Hart, Lynette A. Hart, Abigail P. Thigpen, Neil H. Willits

**Affiliations:** ^1^Department of Anatomy, Physiology and Cell Biology, School of Veterinary Medicine, University of California, Davis, Davis, CA, United States; ^2^Department of Population Health and Reproduction, School of Veterinary Medicine, University of California, Davis, Davis, CA, United States; ^3^Department of Statistics, University of California, Davis, Davis, CA, United States

**Keywords:** spay, neuter, hip dysplasia, cranial cruciate ligament, elbow dysplasia

## Abstract

The early neutering of male and female dogs and its relationship to an increased risk of joint disorders and some cancers has recently become a concern, raising questions about the standard practice in the U.S. and much of Europe of neutering by 6 months of age. A noteworthy recent finding from this center is that there are major breed differences with small-dog breeds generally showing little vulnerability to neutering compared with breeds of larger body size. These findings on purebreds raise questions for dog owners and veterinarians about mixed-breed dogs. The purpose of this study was to examine a sample of mixed breed dogs of five weight categories using the same veterinary hospital database and diagnostic criteria for joint disorders and cancers as used in the newly published paper on 35 breeds and previous papers on the Golden Retriever, Labrador Retriever, and German Shepherd Dog. The weight categories were <10 kg (739 cases), 10–19 kg (546 cases), 20–29 kg (992 cases), 30–39 kg (604 cases), and over 40 kg (258 cases). Males and females were analyzed separately, as were various ages at neutering. The joint disorders examined were hip dysplasia, cranial cruciate ligament tear or rupture, and elbow dysplasia. The cancers were lymphoma, mast cell tumor, hemangiosarcoma, and osteosarcoma. There was no significant increased occurrence of one or more cancers, compared with intact dogs, in any weight category. However, in the three categories of dogs weighing 20 kg or more, neutering before 1 year generally was significantly associated with risks of one or more joint disorders above that of dogs left intact, commonly to 3 times the level of intact dogs, with sex differences in the degrees of joint disorders associated with neutering. For the dogs in the two weight categories <20 kg, no increased risks were found for joint disorders. This information can be useful to dog caregivers in deciding on the age at which to neuter specific dogs, and for veterinarians offering guidance to pet owners.

## Introduction

Neutering male and spaying female dogs (both referred to as neutering) at or before 6 months of age has become routine in the US and much of Europe (1). In the US several states require neutering of all dogs before being released for adoption even if this is well before 6 months of age.

In the meanwhile, investigations have revealed that joint disorders and some cancers may increase in association with early neutering. For example, one study found that hip dysplasia and cranial cruciate ligament tears or ruptures were more likely in neutered than intact males and females (2). Another study found that neutering was associated with a 3-fold increase in excessive tibial plateau angle (3), which is a risk factor for cranial cruciate ligament disorders. Among studies on specific breeds are those on the Golden Retriever, Labrador Retriever, and German Shepherd Dog, revealing an increase in the incidence of one or more of the joint disorders with neutering in the first year to 2–4 times the 3–5% incidence in intact dogs (4–6).

Certain cancers are also known to be more likely in neutered than intact dogs. The occurrence of lymphoma was found to be higher in spayed than intact females (7), as was the occurrence of mast cell tumors (8) and hemangiosarcoma (9). In Golden Retriever females spaying at any age tested, up through 8 years of age, increased the risk of one or more of the cancers by 2–4% (5). A study of over 40,000 dogs utilizing the Veterinary Medical Database found that neutered males and females were more likely to die of cancer than intact dogs (10).

Neutering appears to even have an effect on brain function. A study from 2001 on older male dogs revealed that neutering males was associated accelerated signs of age-related cognitive dysfunction (11). A recent finding along these lines was that the absence of estrogen due to spaying was associated with accelerated brain aging in females (12).

Another paper gives data on increased risks of joint disorders and some cancers associated with neutering in some 35 breeds of dogs, and reveals wide breed-specific variability with some breeds having little vulnerability to neutering and other breeds rather pronounced vulnerability (13). Given that the majority of dogs adopted are of mixed breeds, the goal of this study was to derive data-based information for mixed breed dogs for guidance on the best age to neuter to avoid increasing the risks of joint disorders and cancers typically associated with neutering for those wishing to neuter adopted puppies. The intention was to use the same veterinary hospital database and diagnostic criteria for the diseases as was used with the published studies on the retrievers and German Shepherd Dogs and the paper on 35 breeds of dogs.

## Methods

### Ethics Statement

No animal care and use approval was required because, in conformity with the campus policy, faculty of the University of California-Davis, School of Veterinary Medicine, are allowed use of the record system for research purposes. Strict confidentiality of the owners and their dogs was maintained.

### Subjects

To handle the wide range of body sizes, and to address the issue that body size may influence vulnerability to neutering, five weight categories were established. These were: Small <10 kg (<22 lbs.), Medium 10–19 kg (22–42 lbs.), Standard 20–29 kg (43–64 lbs.), Large 30–39 kg (65–86 lbs.), and Giant 40+ kg (87+ lbs.).

### Study Parameters

The computerized record system of the Veterinary Medical Teaching Hospital (VMTH) at the University of California, Davis, with over 50,000 new cases currently per year, dating back 20 years, was used for the database. The occurrence of joint disorders, namely hip dysplasia (HD), cranial cruciate ligament tears or rupture (CCL), and elbow dysplasia (ED) were examined the same as in previous studies on the retrievers and German Shepherd Dog (4–6). Typically there were signs of lameness, difficulty in moving, and/or joint pain, and diagnosis was confirmed by orthopedic examination, radiographic evidence, and/or surgery. The diagnosis was made at the VMTH, or by the referral hospital and confirmed at the VMTH.

Records were also examined for the occurrence of cancers—those that had been reported to be increased with neutering, namely lymphoma (LSA), hemangiosarcoma (HSA), mast cell tumor (MCT), and osteosarcoma (OSA). For females, mammary cancer (MC), pyometra (PYO), and urinary incontinence (UI) also were tracked. The diagnosis of cancers was based on the presence of a tissue mass, lumps on the skin, or enlarged lymph nodes, and confirmed by appropriate blood cell analyses, chemical panels, histopathology, cytology, and/or imaging. PYO was confirmed by ultrasonic evidence and/or after removal of the uterus. UI was confirmed by frequent urination, urinalyses, and exclusion of urinary tract infection and/or other disease. If a diagnosis was listed in the record as “suspected,” the case was excluded from the analysis for that specific disease. Prostate cancer in males, sometimes considered as comparable to mammary cancer, was not tracked because of the low incidence- −0.35% (14). If the cancer did occur more frequently, it would have been interesting to follow because the cancer tends to increase in incidence with neutering (14).

Joint disorders, cancers including MC, PYO, and UI were examined with regard to dogs neutered at <6 mo., 6–11 mo., 1 year (12–23 mo.), or 2–8 years, or left intact. Appointments were tracked through 11 years of age. Because the median age of diagnosis of MC is about 10 years (15) tracking dogs through 11 years of age would be insufficient to catch many cases even if the case record had information extending to that age.

The case records of neutered dogs that developed a disease of interest were examined to confirm that the dog was neutered prior to the diagnosis or signs of the disease. If the dog developed signs of the disease prior to neutering, the dog was considered intact for that disease. However, it was still listed as neutered for any disease that occurred after neutering. If a disease of interest occurred before 12 months of age, the dog was removed for that disease analysis, but included in analyses of other diseases. The number of cases for various diseases therefore varied in the final analyses for different diseases.

In most instances, neutering age was not included in the records, and the cases were not included in the analyses. Of course, with the intact dogs, this was not an issue and this meant there were proportionately more intact cases in each weight category than would be expected in the general population. However, the specific proportions of intact or neutered dogs with a disease were not affected by the overrepresentation of intact dogs.

With regard to the data on joint disorders, there is the possible effect of body weight, as manifested in body condition score (BCS), because neutering is recognized as predisposing dogs to increased BCS and the occurrence of joint disorders (16). In previous papers (4–6) we found that among neutered dogs, the occurrence of joint disorders was not related to BCS; in the current paper BCSs are not reported.

### Statistical Analyses

In the neutered and intact groups, survival analysis was used to test for differences with respect to the hazard of a joint disorder or cancer while adjusting for the differences in time at risk for a disease. *Post hoc* comparisons among the subgroups were based on least squares means of the hazard within each subgroup. The groups were compared initially using a Kaplan Meier life table analysis. Where the Kaplan Meier test showed significance at the *p* < 0.05 level, both the log-rank and Wilcoxon tests were used for further analyses. Joint disorders are expected to be seen at a similar risk throughout a dog's lifespan, so the log-rank test was used initially. If the log-rank test did not show significance but the Wilcoxon test did, the Wilcoxon test result was reported with significance level and marked by an asterisk. The reverse rule of thumb was used with cancers. For all statistical tests, two-tailed levels of significance were set at *p* < 0.05 and reported as either *p* < 0.05 or *p* < 0.01. Each weight group and sex was analyzed separately, and the *P*-values are reported in the tables for weight categories in Appendix 1.

## Results

A short paragraph below for each weight group summarizes the main findings on joint disorders and cancers for both males and females, and MC, PYO, and UI for females. Also included is a summary, for those wishing to neuter, with a suggested guideline for neutering ages for males and females to avoid increasing the risks of a disease under consideration.

In Appendix 1, each weight group is represented on a separate page and the numbers of intact and neutered males and females are given. In the Appendix, the percentages of dogs with each of the diseases are given for intact males and intact females as well as those neutered at various age ranges. Statistical analyses compared the occurrences of joint disorders and cancers between intact dogs and dogs of each neuter age. If the comparison was significant at either the *p* < 0.05 or *p* < 0.01 level, the data were bolded and the *p*-value was given. The detailed datasets are available online (Figshare, doi: 10.6084/m9.figshare.7231010).

Table 1 is a very brief summary of spaying and neutering guidelines based on findings regarding joint disorders and cancers for each weight group. In Appendix 2 the mean age of last entry in the record for dogs in each weight group is given. Across all weight groups within the study range the mean age of last entry for intact males was 6.0 (range 5.13–6.73), for neutered males was 5.0 years (range 4.19–6.08), for intact females 4.9 (range 4.32–6.65), and for neutered females 5.1 years (range 4.19–6.08).

**Table 1:** Suggested guidelines for age of neutering for five mixed breed weight groups.

**Suggested Guidelines for Age of Neutering: 5 Mixed Breed Groups**								

	**Males**				**Females**			
	**Leave intact**	**Choice**	**Beyond 11 months**	**Beyond 23 months**	**Leave intact**	**Choice**	**Beyond 11 months**	**Beyond 23 months**
Small Medium Standard Large Giant		✓ ✓	✓ ✓	✓		✓ ✓	✓ ✓ ✓^*^	

* *Consider neutering beyond 11 months due to weight*.

*Summary of spaying and neutering guidelines based on findings regarding joint disorders and cancers. The term “choice” means there was no increased risk for any age of neutering*.

### General Findings

In the five weight categories of mixed breed dogs, those weighing 20 kg and above had significant increases in joint disorders with early neutering. For those left intact, the occurrence of a joint disorder ranged in the Standard dogs from 1 and 4% for males and females, respectively, to 9 and 17% in the Giant male and female dogs, respectively. For Standard dogs neutered in the first year, the occurrence of joint disorders ranged from 5% and up to 12% in male and female dogs, respectively. It rose to 28% in Giant male dogs neutered at <6 months but did not increase in the Giant female dogs. In the two weight groups below 20 kg there was no significant increase in incidence of joint disorders above that of the intact dogs.

Of the cancers followed, the occurrence in intact dogs reached as high as 15% (higher in the heavier weight categories of dogs). There were no evident increases with neutering of males or females in any age and weight group, however the occurrences of cancers merit vigilance by the caregivers. The occurrences of MC, PYO, and UI in females are listed for each weight group. The following are brief summaries for each of the five weight categories.

### Small <10 kg (<22 lbs.)

The study population was 152 intact males, 201 neutered males, 148 intact females, and 238 spayed females for a total of 739 cases. Just one intact male had a joint disorder, and no joint disorders were reported in neutered males. For intact females, 2% had a joint disorder, with no evident increase with spaying. For intact males, just 1% had a cancer and neutering was not associated with any evident increase. For intact females, 4% had a cancer and with spaying there was no increase in cancers above that of intact females. In intact females, 6% were diagnosed with MC as were 5% of those spayed at 2–8 years. PYO was diagnosed in 3% of intact females. In early-spayed females no UI was reported. Lacking a noticeable occurrence of increased joint disorders or cancers with neutering, those wishing to neuter a male or a female should decide on the appropriate age.

### Medium 10–19 kg (22–42 lbs.)

The study population was 94 intact males, 114 neutered males, 90 intact females, and 248 spayed females for a total of 546 cases. Just one intact and one neutered male had a joint disorder. In intact females, 5% had a joint disorder with no increase with spaying. For cancers in intact males, 7% had a cancer with no increase with neutering. For intact females, 2% had cancer with no increase with spaying. In intact females, 7% were diagnosed with MC, as were 4% of those spayed at 2–8 years. The occurrence of PYO was diagnosed in 5% of intact females. UI was diagnosed in 4–6% of females spayed at <6 mo. through 1 year. Lacking a noticeable occurrence of increased joint disorders or cancers with neutering, those wishing to neuter a male or female should decide on the appropriate age.

### Standard 20–29 kg (43–64 lbs.)

The study population was 154 intact males, 257 neutered males, 129 intact females, and 452 spayed females for a total of 992 cases. This is the size category of Golden Retrievers, Labrador Retrievers, and German Shepherd Dogs. Of intact males, 3% had a joint disorder. At neutering periods <6mo. and 6–11 mo., the occurrences rose to a significant 5% (*p* < 0.05 and *p* < 0.01 respectively). In intact females, 4% had a joint disorder, but at spay intervals of <6 mo. and 6–11 mo., the occurrences rose significantly to 10 and 12 percent (*p* < 0.05 and *p* < 0.01, respectively). There was no increase in joint disorder occurrence with neutering beyond 12 months of age in either sex. The occurrence of cancers followed for intact males and females was 3%, and this was not noticeably increased with neutering at any age. In intact females, 4% were diagnosed with MC and 5% were diagnosed with PYO. Among females spayed at 2–8 years, 2% had MC. UI was diagnosed in 3% of females spayed at <12 months. For those wishing to neuter, the suggested guideline for both males and females, given the risks of joint disorders in those neutered early, is to delay neutering to 12 months of age or beyond.

### Large Mixed Breed 30–39 kg (65–86 lbs.)

The study population was 176 intact males, 196 neutered males, 57 intact females, and 175 spayed females for a total of 604 cases. Of intact males, 8% had a joint disorder. This occurrence was significantly increased with neutering at <6 mo. to 17% (*p* < 0.01) and to 11% with neutering at 6–11 mo. (*p* < 0.01, combined with <6 mo.). With intact females, the occurrence of joint disorders was 0 percent, but with spaying at <6 mo. this level was significantly increased to 10% (*p* < 0.05) and at 6–11 mo. to 23% (*p* < 0.01) The occurrence of cancers followed for intact males was 15%—higher than the neuter groups but not a significant difference. Similarly, in intact females, this measure was 13%, and higher than any spay group, but not significantly differing. In intact females, 2% were diagnosed with MC, and 4% of females spayed at 2–8 years had MC. PYO was diagnosed in 7% of intact females. UI was diagnosed in 9% of females spayed at <6 mo., a non-significant increase over the 0% of intact females. For those wishing to neuter, the suggested guideline for both males and females, given the risks of joint disorders in those neutered early, is to delay neutering to 12 months of age or beyond. This also avoids the vulnerability to UI in early-spayed females.

### Giant Mixed Breed 40+ kg (87+ lbs.)

The study population was 88 intact males, 107 neutered males, 17 intact females, and 46 spayed females for a total of 258 cases. Of intact males, 9% had a joint disorder. Neutering at <6 mo. was associated with a significant, 3-fold, increase to 28% (*p* < 0.01), and the significant increase continued through neutering at 6–11 mo. (*p* < 0.05) and 1 year (*p* < 0.05), at the 11% level still higher than the intacts. In intact females, 17% had a joint disorder, an occurrence that did not significantly increase with spaying. The occurrence of cancers for intact males was 10% and for intact females, 6%. There were no significant increases in this measure for either sex with neutering at any age. In intact females, none was diagnosed with MC, although PYO was diagnosed in 16%. Of females spayed at <6 mo., UI occurred in 9%. The suggested guideline for males, for those wishing to neuter, given the marked occurrence of joint disorders at neuter periods through 1 year, is to delay neutering until the male is 2 years of age. Those wishing to spay a female should decide on the appropriate age; keeping in mind the large body size and age of musculoskeletal maturation; it is suggested to wait until 1 year.

## Discussion

One major finding from the present study is that despite the dogs being of mixed breeds, there was uniformity in the relationship of body weight and association of neutering with increased risks of joint disorders. The cutoff of 20 kg, where dogs above this point are significantly at risk for joint disorders, but those below this cutoff are not of significant increased risk, offers those adopting puppies, and wishing to neuter, some useful guidelines for ages of neutering with regard to avoiding the increased risk of painful and disabling joint disorders.

There was no clear picture with the cancers followed, undoubtedly reflecting the diversity of breeds involved in mixed breed dogs and the breed-specific differences with regard to vulnerability to different cancers.

This study focuses primarily on dogs going up through their middle-age years, and presents extensive data on diseases having early onsets, prior to dogs' elderly ages. A limitation is that the study has little data going into the second decade of canine life when diseases such as mammary cancer become more frequent.

For those adopting a mixed breed puppy from a shelter or other source, the data in this study suggest that the risks of a joint disorder following early neutering can be predicted on the basis of the dog's body weight. Those that are expected to reach at least 20 kg as adults have a significantly increased risk of one or more joint disorders that is up to 20% above the level of intact dogs. The guidelines for age of neutering are offered in the Results Section above. If the mixed breed puppy is partially of an identifiable breed, such as Labrador Retriever, one could check the breed for cancers as well as joint disorders for guidance regarding the age for neutering. Pet owners and veterinarians can consider this information when making decisions or recommendations regarding the age for neutering a dog that will be in a specific adult weight category.

## Data Availability Statement

The datasets presented in this study can be found in online repositories. The names of the repository/repositories and accession number(s) can be found below: Figshare, doi: 10.6084/m9.figshare.7231010.

## Ethics Statement

Ethical review and approval was not required for the animal study because no approval was required to use hospital records. Written informed consent for participation was not obtained from the owners because it was not needed for hospital records.

## Author Contributions

BH, LH, and AT: conceived and designed study. AT, BH, and LH: collected and complied and analyzed data. NW: statistical analyses. BH, LH, AT, and NW: drafted and edited manuscript. All authors contributed to the article and approved the submitted version.

## Conflict of Interest

The authors declare that the research was conducted in the absence of any commercial or financial relationships that could be construed as a potential conflict of interest.
