# Supplementary material for: Assisting Decision-Making on Age of Neutering for Mixed Breed Dogs of Five Weight Categories: Associated Joint Disorders and Cancers
Source: Front Vet Sci. 2020 Jul 31;7:472. doi: 10.3389/fvets.2020.00472 (PMC7412743; doi:10.3389/fvets.2020.00472)
Supplement: Supplementary file 1 [file Data_Sheet_1.pdf]

Appendix 1. Results for Five Weight Categories of Mixed Breed Dogs

Small < 10 kg. (< 22 lbs.)
The study population was 152 intact males, 201 neutered males, 148 intact females, and 238 spayed females for a total of 739 cases.

Joint disorders: hip dysplasia (HD), cranial cruciate ligament rupture (CCL), and elbow dysplasia (ED). Just one intact male had a joint disorder, and no joint disorders were reported in neutered males. For intact females, 2 percent had a joint disorder, and for those spayed beyond 6 months, there was a 2-3 percent occurrence.

Cancers: lymphoma (LSA), mast cell tumor (MCT), hemangiosarcoma (HSA), and osteosarcoma (OSA). For intact males, just 1 percent had a cancer and at neuter intervals 6-11 mo. and 2-8 years, 1-2 percent were diagnosed with a cancer followed. For intact females, 4 percent had a cancer and just 2 percent of those spayed at 6-11 months were diagnosed with a cancer.

Mammary Cancer (MC) in Females. In intact females, 6 percent were diagnosed with MC as were 5 percent of those spayed at 2-8 years.

Pyometra (PYO) in Females. PYO was diagnosed in 3 percent of intact females.

Urinary Incontinence (UI) in Females. In early-spayed females, no UI was reported.

Bottom line. Lacking a noticeable occurrence of increased joint disorders or cancers with neutering, those wishing to neuter a male or female should select the appropriate age.

Joint disorders. For ages 1 through 11 years and for each neuter period.

	HD	CCL	ED	At Least One
Male < 6 months	0/24 (0)	0/24 (0)	0/24 (0)	0/24 (0)
Male 6 – 11 months	0/52 (0)	0/50 (0)	0/52 (0)	0/50 (0)
Male 1 year	0/51 (0)	0/51 (0)	0/51 (0)	0/51 (0)
Male 2 – 8 years	0/70 (0)	0/70 (0)	0/70 (0)	0/70 (0)
Male Intact	0/149 (0)	1/149 (0.67)	0/151 (0)	1/147 (0.68)
Female < 6 months	0/30 (0)	0/30 (0)	0/30 (0)	0/30 (0)
Female 6 – 11 months	0/63 (0)	1/65 (1.54)	1/65 (1.54)	2/64 (3.13)
Female 1 year	0/50 (0)	1/51 (1.96)	0/51 (0)	1/50 (2)
Female 2 – 8 years	1/81 (1.23)	1/78 (1.28)	0/81 (0)	2/78 (2.56)
Female Intact	0/148 (0)	3/149 (2.01)	0/148 (0)	3/149 (2.01)

Cancers. For ages 1 through 11 years and for each neuter period.

	LSA	MCT	HSA	OSA	At Least One
Male < 6 months	0/24 (0)	0/24 (0)	0/24 (0)	0/24 (0)	0/24 (0)
Male 6 – 11 months	0/51 (0)	1/52 (1.92)	0/51 (0)	0/52 (0)	1/50 (2)
Male 1 year	0/49 (0)	0/51 (0)	0/51 (0)	0/51 (0)	0/49 (0)
Male 2 – 8 years	0/69 (0)	1/70 (1.43)	0/69 (0)	0/70 (0)	1/69 (1.45)
Male Intact	1/147 (0.68)	0/152 (0)	0/151 (0)	0/150 (0)	1/145 (0.69)
Female < 6 months	0/28 (0)	0/28 (0)	0/30 (0)	0/30 (0)	0/27 (0)
Female 6 – 11 months	0/65 (0)	1/65 (1.54)	0/65 (0)	0/65 (0)	1/65 (1.54)
Female 1 year	0/49 (0)	0/51 (0)	0/51 (0)	0/51 (0)	0/49 (0)
Female 2 – 8 years	0/78 (0)	0/81 (0)	0/81 (0)	0/80 (0)	0/78 (0)
Female Intact	2/142 (1.41)	3/145 (2.07)	0/146 (0)	0/148 (0)	5/138 (3.62)

Medium 10-19 kg. (22-42 lbs.)
The study population was 94 intact males, 114 neutered males, 90 intact females, and 248 spayed females for a total of 546 cases.

Joint disorders: hip dysplasia (HD), cranial cruciate ligament rupture (CCL), and elbow dysplasia (ED). Just one intact male and one neutered at 2-8 years had a joint disorder. In intact females, 5 percent had a joint disorder, as did 2-3 percent of those spayed from < 6 mo. through 1 year of age.

Cancers: lymphoma (LSA), mast cell tumor (MCT), hemangiosarcoma (HSA), and osteosarcoma (OSA). For intact males, 7 percent had a cancer and for those neutered at 1 year and 2-8 years, there was just one occurrence in each category. For intact females, 2 percent had cancer and there were just two cases with spaying ages of 6-11 mo. through 2-8 years.

Mammary Cancer (MC) in Females. In intact females, 7 percent were diagnosed with MC, as were 4 percent of those spayed at 2-8 years

Pyometra (PYO) in Females. The occurrence of PYO was diagnosed in 5 percent of intact females.

Urinary Incontinence (UI) in Females. UI did not occur in intact females but was diagnosed in 4 to 6 percent of females spayed at < 6 mo. through 1 year.

Bottom line. Lacking a noticeable occurrence in males or females of increased joint disorders or cancers with neutering, those wishing to neuter should select the appropriate age.

Joint disorders. For ages 1 through 11 years and for each neuter period.

	HD	CCL	ED	At Least One
Male < 6 months	0/15 (0)	0/15 (0)	0/15 (0)	0/15 (0)
Male 6 – 11 months	0/17 (0)	0/17 (0)	0/17 (0)	0/17 (0)
Male 1 year	0/39 (0)	0/38 (0)	0/39 (0)	0/38 (0)
Male 2 – 8 years	0/35 (0)	1/34 (2.94)	0/35 (0)	1/34 (2.94)
Male Intact	0/93 (0)	1/92 (1.09)	0/94 (0)	1/92 (1.09)
Female < 6 months	0/34 (0)	1/33 (3.03)	0/33 (0)	1/32 (3.13)
Female 6 – 11 months	0/59 (0)	1/59 (1.69)	0/61 (0)	1/59 (1.69)
Female 1 year	2/87 (2.3)	3/87 (3.45)	0/88 (0)	3/87 (3.45)
Female 2 – 8 years	0/58 (0)	0/58 (0)	0/59 (0)	0/58 (0)
Female Intact	2/89 (2.25)	3/88 (3.41)	1/90 (1.11)	4/88 (4.55)

Cancers. For ages 1 through 11 years and for each neuter period.

	LSA	MCT	HSA	OSA	At Least One
Male < 6 months	0/15 (0)	0/15 (0)	0/15 (0)	0/15 (0)	0/15 (0)
Male 6 – 11 months	0/17 (0)	0/16 (0)	0/17 (0)	0/17 (0)	0/16 (0)
Male 1 year	0/39 (0)	1/39 (2.56)	0/39 (0)	0/39 (0)	1/39 (2.56)
Male 2 – 8 years	1/35 (2.86)	0/34 (0)	0/35 (0)	0/35 (0)	1/34 (2.94)
Male Intact	2/92 (2.17)	5/96 (5.21)	0/94 (0)	0/94 (0)	7/94 (7.45)
Female < 6 months	0/32 (0)	0/34 (0)	0/34 (0)	0/34 (0)	0/32 (0)
Female 6 – 11 months	0/58 (0)	1/60 (1.67)	0/61 (0)	0/61 (0)	1/57 (1.75)
Female 1 year	0/86 (0)	1/84 (1.19)	0/88 (0)	0/88 (0)	1/83 (1.2)
Female 2 – 8 years	0/58 (0)	0/57 (0)	0/58 (0)	0/59 (0)	0/55 (0)
Female Intact	2/87 (2.3)	0/90 (0)	0/90 (0)	0/90 (0)	2/87 (2.3)

Standard 20-29 kg (43-64 lbs.)

The study population was 154 intact males, 257 neutered males, 129 intact females, and 452 spayed females for a total of 992 cases.

Joint disorders: hip dysplasia (HD), cranial cruciate ligament rupture (CCL), and elbow dysplasia (ED). Of intact males, 3 percent had a joint disorder. At neutering periods < 6m. and at 6-11 mo., this occurrence rose significantly to 5 percent ($p < 0.05$* and $p < 0.01$*, respectively). In intact females, 4 percent had a joint disorder, but at spay intervals of < 6 mo. and 6-11 mo., the occurrence rose significantly to 10 and 12 percent ($p < 0.05$ and $p < 0.01$, respectively) with no increase in occurrence beyond 12 months.

Cancers: lymphoma (LSA), mast cell tumor (MCT), hemangiosarcoma (HSA), and osteosarcoma (OSA). The occurrence of cancers for intact males and females was 3 percent, and this was not noticeably increased with neutering at any age.

Mammary Cancer (MC) in Females. In intact females, 4 percent were diagnosed with MC, and 2 percent of females spayed at 2-8 years had MC.

Pyometra (PYO) in Females. UI was in 3 percent of females spayed by 11 months.

Urinary Incontinence (UI) in Females. UI was diagnosed in 3 percent of females spayed at less than 11 months.

Bottom line. The suggested guideline for both males and females, given the risks of joint disorders in those neutered early, is to delay neutering to 12 months or beyond.

Joint Disorders. For ages 1 through 11 years and for each neuter period. Bold values indicate significance over the intact group. The asterisk (*) indicates when there was significance using the Wilcoxon test, but no significance using the log-rank test. The dagger (†) indicates significance when the early groups (< 6 mo. and 6-11 mo.) are combined.

	HD	CCL	ED	At Least One
Male < 6 months	0/46 (0)	2/46 (4.35)	0/47 (0)	**2/41 (4.88)* †**
Male 6 – 11 months	3/61 (4.92)	0/65 (0)	0/64 (0)	**3/61 (4.92)* †**
Male 1 year	0/65 (0)	1/67 (1.49)	0/68 (0)	1/64 (1.56)
Male 2 – 8 years	1/60 (1.67)	0/62 (0)	0/63 (0)	1/59 (1.69)
Male Intact	4/157 (2.55)	0/152 (0)	0/153 (0)	4/154 (2.6)
Female < 6 months	2/81 (2.47)	4/93 (4.3)	2/87 (2.3)	**8/79 (10.13) †**
Female 6 – 11 months	9/134 (6.72)	9/135 (6.67)	1/137 (0.73)	**16/130(12.31)†**
Female 1 year	0/94 (0)	2/94 (2.13)	1/94 (1.06)	2/93 (2.15)
Female 2 – 8 years	0/112 (0)	0/114 (0)	1/117 (0.85)	1/109 (0.92)
Female Intact	3/128 (2.34)	2/129 (1.55)	1/129 (0.78)	5/129 (3.88)

Cancers. For ages 1 through 11 years and for each neuter period.

	LSA	MCT	HSA	OSA	At Least One
Male < 6 months	0/48 (0)	1/49 (2.04)	0/48 (0)	0/49 (0)	1/48 (2.08)
Male 6 – 11 months	1/62 (1.61)	0/65 (0)	0/65 (0)	0/64 (0)	1/61 (1.64)
Male 1 year	1/68 (1.47)	1/68 (1.47)	0/68 (0)	0/67 (0)	2/67 (2.99)
Male 2 – 8 years	0/63 (0)	0/60 (0)	0/63 (0)	1/63 (1.59)	1/60 (1.67)
Male Intact	2/154 (1.3)	1/154 (0.65)	1/153 (0.65)	1/153 (0.65)	5/152 (3.29)
Female < 6 months	0/92 (0)	1/93 (1.08)	2/93 (2.15)	0/94 (0)	3/91 (3.3)
Female 6 – 11 months	1/138 (0.72)	1/138 (0.72)	3/139 (2.16)	1/139 (0.72)	6/137 (4.38)
Female 1 year	1/93 (1.08)	0/95 (0)	0/95 (0)	0/95 (0)	1/93 (1.08)
Female 2 – 8 years	0/115 (0)	1/117 (0.85)	0/115 (0)	0/117 (0)	1/113 (0.88)
Female Intact	2/128 (1.56)	1/129 (0.78)	1/130 (0.77)	0/128 (0)	4/128 (3.13)

Large 30-39 kg (65-86 lbs.)
The study population was 176 intact males, 196 neutered males, 57 intact females, and 175 spayed females for a total of 604 cases.

Joint disorders: hip dysplasia (HD), cranial cruciate ligament rupture (CCL), and elbow dysplasia (ED). Of intact males, 8 percent had a joint disorder. This occurrence was significantly increased with neutering at less than 6 mo. to 17 percent ($p< 0.01$). With intact females, the occurrence of joint disorders was 0 percent, but with spaying at < 6 mo. this level was increased to 10 percent and at 6-11 mo., to 23 percent; both increases were significant ($p< 0.05$ and $p< 0.01$).

Cancers: lymphoma (LSA), mast cell tumor (MCT), hemangiosarcoma (HSA), and osteosarcoma (OSA). The occurrence of cancers for intact males was 15 percent – higher than in any neuter group. In intact females, this measure was 13 percent --- higher than any spay group. None of these differences was significant, for males or females.

Mammary Cancer (MC) in Females. In intact females, 2 percent were diagnosed with MC, and 4 percent of females spayed at 2-8 years had MC.

Pyometra (PYO) in Females. PYO was diagnosed in 7 percent of intact females.

Urinary Incontinence (UI) in Females. UI was diagnosed in 9 percent of females spayed at < 6 mo., a non-significant increase over the 0 percent of intact females.

Bottom line. The suggested guideline for both males and females, given the risks of joint disorders in those neutered early, is to delay neutering to 12 months of age or beyond. This also avoids the possible vulnerability to UI in early-spayed females.

Joint Disorders. For ages 1 through 11 years and for each neuter period. Bold values indicate significance over the intact group. The dagger (†) indicates significance over the intact group when the early groups (< 6 mo. and 6-11 mo.) are combined.

	HD	CCL	ED	At Least One
Male < 6 months	2/41 (4.88)	6/45 (13.33)	0/43 (0)	**7/41 (17.07)** †
Male 6 – 11 months	2/38 (5.26)	3/46 (6.52)	0/45 (0)	**4/37 (10.81)** †
Male 1 year	1/50 (2)	0/51 (0)	1/51 (1.96)	1/50 (2)
Male 2 – 8 years	0/44 (0)	0/46 (0)	1/48 (2.08)	1/45 (2.22)
Male Intact	7/173 (4.05)	5/177 (2.82)	4/174 (2.3)	13/174 (7.47)
Female < 6 months	1/31 (3.23)	3/32 (9.38)	0/33 (0)	**3/30 (10)** †
Female 6 – 11 months	4/50 (8)	8/48 (16.67)	1/51 (1.96)	**11/47 (23.4)** †
Female 1 year	0/35 (0)	1/37 (2.7)	0/38 (0)	1/34 (2.94)
Female 2 – 8 years	0/50 (0)	2/50 (4)	0/51 (0)	2/48 (4.17)
Female Intact	0/55 (0)	0/57 (0)	0/57 (0)	0/55 (0)

Cancers. For ages 1 through 11 years and for each neuter period.

	LSA	MCT	HSA	OSA	At Least One
Male < 6 months	2/42 (4.76)	0/43 (0)	0/45 (0)	0/45 (0)	2/42 (4.76)
Male 6 – 11 months	2/46 (4.35)	0/44 (0)	2/45 (4.44)	0/46 (0)	4/44 (9.09)
Male 1 year	0/51 (0)	1/51 (1.96)	1/51 (1.96)	1/51 (1.96)	3/51 (5.88)
Male 2 – 8 years	1/48 (2.08)	1/47 (2.13)	0/48 (0)	0/48 (0)	2/47 (4.26)
Male Intact	13/175 (7.43)	6/176 (3.41)	1/174 (0.57)	5/174 (2.87)	25/172 (14.53)
Female < 6 months	1/32 (3.13)	2/33 (6.06)	0/33 (0)	1/33 (3.03)	4/32 (12.5)
Female 6 – 11 months	0/50 (0)	2/50 (4)	1/51 (1.96)	0/50 (0)	3/48 (6.25)
Female 1 year	1/38 (2.63)	1/37 (2.7)	1/37 (2.7)	0/38 (0)	3/37 (8.11)
Female 2 – 8 years	0/51 (0)	2/52 (3.85)	0/52 (0)	1/52 (1.92)	3/51 (5.88)
Female Intact	5/56 (8.93)	1/57 (1.75)	0/56 (0)	1/57 (1.75)	7/55 (12.73)

Giant 40+ kg (87+ lbs.)

The study population was 88 intact males, 107 neutered males, 17 intact females, and 46 spayed females for a total of 258 cases.

Joint disorders: hip dysplasia (HD), cranial cruciate ligament rupture (CCL), and elbow dysplasia (ED). Of intact males, 9 percent had a joint disorder. Neutering at < 6 mo. was associated with a significant increase to 28 percent ($p < 0.01$), and the significant increase continued through neutering at 6-11 mo. ($p < 0.05$*) and 1 year ($p < 0.05$). In the small group of 17 intact females, 17 percent had a joint disorder, an occurrence that did not significantly increase with spaying, even at < 6 mo. (18 percent).

Cancers: lymphoma (LSA), mast cell tumor (MCT), hemangiosarcoma (HSA), and osteosarcoma (OSA). The occurrence of cancers for intact males was 10 percent and for females, 6 percent. There were no significant increases in this measure with spaying at any age, although the 14 percent occurrence in females at the 2-8 years seems high.

Mammary Cancer (MC) in Females. No intact females, were diagnosed with MC.

Pyometra (PYO) in Females. In intact females, PYO was diagnosed in 16 percent.

Urinary Incontinence (UI) in Females. UI occurred in 9 percent spayed at < 6 mo.

Bottom line. The suggested guideline for males, with marked joint disorders at neuter periods through 1 year, is to delay neutering until the male is two years of age. If spaying a female, should decide on the appropriate age, considering the large body size and delayed musculoskeletal development. It is suggested to delay neutering until 12 months or beyond.

Joint disorders. For ages 1 through 11 years and for each neuter period. Bold values indicate significance over intacts. Asterisk (*) shows significance using the Wilcoxon test: no significance with log-rank test. Dagger (†) indicates significance over intacts when the early groups are combined.

	HD	CCL	ED	At Least One
Male < 6 months	2/17 (11.76)	5/18 (27.78)	0/18 (0)	**5/18 (27.78)** †
Male 6 – 11 months	1/27 (3.7)	2/28 (7.14)	0/27 (0)	**3/27 (11.11)*** †
Male 1 year	0/20 (0)	1/19 (5.26)	2/21 (9.52)	**2/19 (10.53)**
Male 2 – 8 years	0/36 (0)	0/32 (0)	0/36 (0)	0/32 (0)
Male Intact	1/86 (1.16)	7/91 (7.69)	0/86 (0)	8/87 (9.2)
Female < 6 months	1/11 (9.09)	1/12 (8.33)	0/11 (0)	2/11 (18.18)
Female 6 – 11 months	0/10 (0)	0/12 (0)	0/11 (0)	0/10 (0)
Female 1 year	0/4 (0)	0/4 (0)	0/4 (0)	0/4 (0)
Female 2 – 8 years	0/13 (0)	0/13 (0)	0/14 (0)	0/13 (0)
Female Intact	1/18 (5.56)	3/18 (16.67)	0/17 (0)	3/18 (16.67)

Cancers. For ages 1 through 11 years and for each neuter period.

	LSA	MCT	HSA	OSA	At Least One
Male < 6 months	1/18 (5.56)	0/18 (0)	0/17 (0)	0/18 (0)	1/17 (5.88)
Male 6 – 11 months	1/28 (3.57)	2/27 (7.41)	0/27 (0)	0/28 (0)	3/27 (11.11)
Male 1 year	0/20 (0)	1/21 (4.76)	2/21 (9.52)	0/21 (0)	2/21 (9.52)
Male 2 – 8 years	1/36 (2.78)	0/36 (0)	0/34 (0)	1/35 (2.86)	2/33 (6.06)
Male Intact	4/84 (4.76)	0/88 (0)	2/87 (2.3)	2/84 (2.38)	8/81 (9.88)
Female < 6 months	1/12 (8.33)	0/12 (0)	0/12 (0)	0/12 (0)	1/12 (8.33)
Female 6 – 11 months	0/12 (0)	0/11 (0)	0/11 (0)	0/12 (0)	0/10 (0)
Female 1 year	0/4 (0)	0/4 (0)	0/4 (0)	0/4 (0)	0/4 (0)
Female 2 – 8 years	1/14 (7.14)	1/14 (7.14)	0/14 (0)	0/14 (0)	2/14 (14.29)
Female Intact	1/17 (5.88)	0/17 (0)	0/17 (0)	0/17 (0)	1/17 (5.88)
