# Supplementary material for: Assisting Decision-Making on Age of Neutering for Mixed Breed Dogs of Five Weight Categories: Associated Joint Disorders and Cancers
Source: Front Vet Sci. 2020 Jul 31;7:472. doi: 10.3389/fvets.2020.00472 (PMC7412743; doi:10.3389/fvets.2020.00472)
Supplement: Supplementary file 2 [file Data_Sheet_2.pdf]

Appendix 2. Mean Age at Last Visit For Mixed Breed Categories. For neutered males (MN), spayed females (FS), intact males (MI), and intact females (FI), the table lists the mean ages at last visit within the study range by weight group. The numbers of neutered and intact females and males for each weight group are also included. The bottom of the table includes the calculation for the overall mean age at last visit within the study range for all dogs included in the study.

	**FS Mean**	**FS No.**	**FI Mean**	**FI No.**	**MN Mean**	**MN No.**	**MI Mean**	**MI No.**
Small, <10 kg	4.89	238	4.85	148	4.19	201	5.13	152
Medium, 10-19 kg	4.89	248	4.94	90	4.98	114	5.97	94
Standard, 20-29 kg	5.04	452	4.32	129	4.79	257	5.78	154
Large, 30-39 kg	5.76	175	5.57	57	5.35	196	6.73	176
Giant, 40+ kg	6.04	46	6.65	17	6.08	107	6.58	88
**OVERALL**	**5.1**	**1159**	**4.9**	**441**	**5.0**	**875**	**6.0**	**664**
